# Association between obstructive sleep apnea severity and endothelial dysfunction in patients with type 2 diabetes

**DOI:** 10.1186/s12933-017-0521-y

**Published:** 2017-03-21

**Authors:** Vanessa Bironneau, François Goupil, Pierre Henri Ducluzeau, Marc Le Vaillant, Pierre Abraham, Samir Henni, Séverine Dubois, Audrey Paris, Pascaline Priou, Nicole Meslier, Claire Sanguin, Wojciech Trzépizur, Ramaroson Andriantsitohaina, Maria Carmen Martinez, Frédéric Gagnadoux

**Affiliations:** 1 Université Bretagne Loire, INSERM UMR 1063, Angers, France; 2 Service de Pneumologie, Centre Hospitalier, Le Mans, France; 3 Unité d’Endocrinologie-Diabétologie-Nutrition, Pole de Médecine, CHRU de Tours, Tours, France; 4 Centre de Recherche Médecine, Sciences, Santé, Santé mentale, Société, CNRS UMR 8211, INSERM UMR U988-EHESS, Villejuif, France; 5 Département de Médecine du Sport et Explorations Fonctionnelles Vasculaires, Université Bretagne Loire, CHU d’Angers, Angers, France; 6 Département d’Endocrinologie, Diabétologie, Nutrition, Université Bretagne Loire, CHU d’Angers, Angers, France; 7 Département de Pneumologie, Université Bretagne Loire, CHU d’Angers, 4 Rue Larrey, 49100 Angers, France; 8 Service d’Endocrinologie, Diabétologie, Centre Hospitalier, Le Mans, France

**Keywords:** Obstructive sleep apnea, Type 2 diabetes, Endothelial function, Peripheral arterial tonometry, Reactive hyperemia index

## Abstract

**Background:**

Obstructive sleep apnea (OSA) and type 2 diabetes (T2D) are associated with endothelial dysfunction a main predictor of late cardiovascular (CV) events. Despite the high prevalence of OSA in patients with T2D, the impact of OSA severity on endothelial function has not been clearly elucidated. The aim of this cross-sectional study was to determine whether increasing OSA severity is associated with poorer endothelial function in patients with T2D.

**Methods:**

140 patients with T2D and no overt CV disease underwent polysomnography, peripheral arterial tonometry, clinic blood pressure (BP) measurement, biological assessment for CV risk factors, daytime sleepiness and health related quality of life (HRQL) questionnaires. The following commonly used cut-offs for apnea-hypopnea index (AHI) were used to define 3 categories of disease severity: AHI < 15 (no OSA or mild OSA), 15 ≤ AHI < 30 (moderate OSA), and AHI ≥ 30 (severe OSA). The primary outcome was the reactive hyperemia index (RHI), a validated assessment of endothelial function.

**Results:**

21.4% of patients had moderate OSA and 47.6% had severe OSA. Increasing OSA severity and nocturnal hypoxemia were not associated with a significant decrease in RHI. Endothelial dysfunction (RHI < 1.67) was found in 47.1, 44.4 and 39.2% of patients with no OSA or mild OSA, moderate OSA and severe OSA, respectively (p = 0.76). After adjustment for confounders including body mass index, increasing OSA severity was associated with higher systolic BP (p = 0.03), lower circulating levels of adiponectin (p = 0.0009), higher levels of sP-selectin (p = 0.03), lower scores in 3 domains of HRQL including energy/vitality (p = 0.02), role functioning (p = 0.01), and social functioning (p = 0.04).

**Conclusions:**

Moderate to severe OSA is very common but has no impact on digital micro-vascular endothelial function in patients with T2D.

## Background

Endothelial dysfunction is a pathophysiological determinant of atherogenesis that occurs at the early stages of coronary artery disease [1] and predicts the occurrence of cardiovascular (CV) events in at-risk subjects [2]. Flow-mediated dilatation (FMD) of the brachial artery has been extensively used as a noninvasive measure of endothelial function. However, the FMD technique is time-consuming and requires a careful learning curve, which limits its routine application in large multicenter studies. Measuring endothelial function by peripheral arterial tonometry (PAT) has recently gained increased attention, as the reactive hyperemia index (RHI) measured by PAT is a validated marker of endothelial function [3]. Validation studies have demonstrated that RHI is correlated with coronary micro-vascular function in patients with early atherosclerosis [4] and predicts CV events [5–7]. Recent study underlined the relationship between immune-inflammatory markers, endothelial and arterial stiffness index in patients with acute ischemic stroke, also suggesting the use of a combination of PAT and arterial stiffness indexes to better categorize patients with ischemic stroke [8, 9]. Nitric oxide (NO) plays an important role in digital reactive hyperemia [10]. Kuvin et al. [11] found a significant correlation (r = 0.55, p < 0.0001) between RHI and FMD in 89 subjects, 38% of whom had a history of coronary artery disease. RHI was also significantly correlated with FMD (r = 0.47; p < 0.001) in non-obese and non-smoker subjects free of overt CV disease [12]. In the Framingham health study, RHI correlated with traditional CV risk factors but the association with FMD was not significant in multivariable-adjusted analyses [13].

The obesity pandemic is associated with an increasing prevalence of type 2 diabetes (T2D) and obstructive sleep apnea (OSA). T2D carries a substantially increased risk of macro and micro-vascular complications, including coronary artery disease, nephropathy, retinopathy and non-healing foot ulcers [14, 15]. Endothelial dysfunction is important in the pathogenesis of diabetic angiopathy due to increased vascular tone, vascular inflammation and oxidative stress [15–17]. Several studies demonstrated that micro-vascular endothelial function assessed by the RHI is impaired in patients with T2D and improved by dietary of pharmacological interventions [15, 18–22]. In a cross-sectional study including 183 patients undergoing coronary angiography, diabetic patients with and without coronary heart disease showed significantly impaired peripheral vascular function assessed by RHI compared to non-diabetic patients without coronary heart disease [18]. A recent study demonstrated that patients with T2D and diabetic foot syndrome have lower mean values of RHI than diabetic subjects without diabetic foot [23]. OSA is also associated with a decrease in endothelial NO synthase and an increase in endothelin-1 levels [24], with hypercoagulability [25], and inflammation [26]. Repeated hypoxemia-reoxygenation episodes induce ROS production [27]. The final result of this process is the development of endothelial dysfunction and micro-vascular impairment [28]. Several clinic-based and population-based studies have demonstrated that OSA is associated with severity-dependent deterioration of endothelial function assessed by RHI in both adults and children [29–34]. Microparticles (MPs) are small plasma membrane vesicles that can be released by a variety of vascular or blood cells and contain both membrane and cytosolic elements. Experimental data has demonstrated that MPs can promote endothelial dysfunction. MPs released in vitro by apoptotic T-lymphocytes impair endothelial function by stimulating oxygen free radical generation, and decreasing Ser1179 phosphorylation of endothelial NO synthase [35]. Recent data suggest that circulating MPs released by activated endothelial cells can be considered as biomarkers of endothelial dysfunction in OSA [35] and in T2D [36].

In clinical practice, OSA is frequently associated with several risk factors for atherosclerosis, including hypertension [37], T2D [38, 39] and the metabolic syndrome [40]. Previous studies demonstrated additive effects on early markers of atherosclerosis in patients with OSA associated with hypertension [41] or the metabolic syndrome [40]. There is evidence to suggest that the presence and severity of untreated OSA is associated with poorer glucose control in patients with T2D [38]. However, although recent data suggested that OSA may be associated with diabetic retinopathy [42], nephropathy [43] and neuropathy [44], there is no conclusive evidence today regarding the impact of the OSA-T2D relation on micro- and macro-vascular diabetic complications [45]. As OSA and T2D often occur concomitantly, the presence of OSA may exert addictive effects on subclinical markers of vascular dysfunction in patients with T2D. The main objective of the present study was to determine whether increasing OSA severity is associated with poorer endothelial function, which could contribute to increase the risk of micro- and macro-vascular complications in patients with T2D and no overt CV disease.

## Methods

### Study design

This study was cross-sectional study done in two French sleep centers (Angers University Hospital and Le Mans General Hospital, France). The study was approved by our local ethical committee (CPP OUEST II, no2011/12). Outcome assessors were unaware of study-group assignment.

### Participants

Patients aged 18–75 with T2D on stable medications for the preceding 3 months, without medical history of CV disease including coronary heart disease, heart failure, arrhythmias and stroke, were recruited from the out-patients diabetes clinics of Angers and Le Mans hospitals. All patients gave their written informed consent to participate in the study.

### Sleep studies

In all patients OSA was assessed by an overnight in-laboratory polysomnography (PSG) (CID 102™, Cidelec, Angers, France) with continuous recording of the following channels: electroencephalogram, electrooculogram, chins electromyogram, arterial oxygen saturation, nasal–oral airflow, electrocardiogram, chest and abdominal wall motion, and body position. Respiratory events were scored manually. An apnea was defined as the complete cessation of airflow and hypopnea as a decrease in the nasal pressure signal of at least 30% in association with either ≥3% arterial oxygen desaturation or an arousal both lasting for at least 10 s [46].

### Clinical evaluation

All clinical examinations and evaluations were conducted during the morning hours following overnight PSG. Clinical evaluation included anthropometric data, smoking habits, medical history, and medication use. Patients who were previously diagnosed as hypertensive and were taking antihypertensive medication were considered as having hypertension. Daytime sleepiness was evaluated by the Epworth sleepiness scale (ESS) [47]. Excessive daytime sleepiness was defined by an ESS ≥ 11. Health related quality of life (HRQL) was evaluated with a validated French-language version of the medical outcomes study 36-item short-form (SF36) [48]. Clinic blood pressure (BP) was measured using a periodically calibrated mercury sphygmomanometer and an appropriate cuff size. Recorded blood pressure was the average of three consecutive readings during a 5-min period following at least 5 min of rest in the sitting position.

### Assessment of micro-vascular endothelial function

Endothelial function was assessed by digital pulse amplitude, using PAT (EndoPAT^®^, Itamar Medical Ltd., Caesarea, Israel) [4]. PAT was performed in the morning following PSG. Patients laid down in a quiet room with probes mounted on both index fingers and a blood pressure cuff around the right arm above the elbow for the hyperemia testing. The left finger was used as the control. Finger arterial pulse wave amplitude was recorded throughout the protocol which included 3 consecutive stages each of 5 min duration: (1) baseline recording; (2) occlusion of the brachial artery by inflating the blood pressure cuff to 50 mm Hg above the baseline systolic pressure and (3) post-occlusion recording after deflation of the cuff to measure of the generated reactive hyperemia response. RHI was calculated as the ratio of the average amplitude of the PAT signal post-to-pre occlusion of the tested arm, normalized to the concurrent signal from the contralateral finger. A RHI value <1.67 indicated endothelial dysfunction [4]. The two participating centers followed the same procedure and PAT assessors were blinded to patient’s study group.

### Biological assessment

Fasting blood samples were taken from the antecubital vein in the morning hours following overnight PSG. Plasma glucose, total serum cholesterol, and high-density lipoprotein serum cholesterol (HDLc) were directly measured in accredited laboratories using standard techniques. Low-density lipoprotein serum cholesterol (LDLc) was calculated. Glycated hemoglobin (HbA1c) was measured in whole blood with ion-exchange high-performance liquid chromatography and used as a clinical indicator of glucose control.

Plasma and serum samples were obtained after centrifugation and stored at −80 °C for assessment of circulating biomarkers of cardiovascular risk. For each ELISA kit, samples were assayed in duplicate. ELISA assays included serum levels of leptin (Human Leptin ELISA KIT, OKAA00022_96 W, AVIVA SYSTEMS BIOLOGY, San Diego, USA; detection limit = 7 pg/ml), adiponectin (Human Adiponectin ELISA KIT, EA2500-1, ASSAYPRO, St Charles, MO; detection limit = 0.3 ng/ml), high-sensitive C-reactive protein (hs-CRP ELISA, DE740011, Demeditec, Germany; detection limit = 0.02 µg/ml), soluble P-selectin (sP-selectin, Human P-Selectin/CD62P, BBE6, R&D Systems Europe, Ltd., UK; detection limit = 0.5 ng/ml), and 8-iso-prostaglandin level (OxiSelect™ 8-iso-Prostaglandin F2α ELISA Kit, STA-337, Cell Biolabs, INC., USA). Inter-assay and intra-assay variation for all the techniques used was <10%. The homeostasis model assessment resistance index (HOMA-IR) was calculated from fasting glucose and insulin concentrations, as follows: insulin (mIU/l) * glucose (mmol/l)/22.5.

In 43 patients circulating microparticles (MPs) were isolated from blood samples within 2 h after completion of sampling, from a peripheral vein using a 21-gauge needle to minimize platelet activation. A first centrifugation of blood was carried out 260*g* for 20 min. Plasma was centrifuged at 1500*g* for 20 min in order to obtain platelet-free plasma. Two hundred microliters of platelet-free plasma were frozen and stored at −80 °C for subsequent analysis. Circulating MPs were quantified and their cellular origin identified using flow cytometry with specific antibodies (Beckman Coulter, Villepinte, France). Anti-CD62P and anti-CD62E antibodies were used to identify P-selectin^+^ and E-selectin^+^ MPs, corresponding to MPs from activated platelets and activated endothelial cells, respectively.

### Statistical analysis

The primary outcome variable was endothelial function assessed by the RHI. Secondary outcomes included clinic BP, biological data and HRQL questionnaires. Characteristics of the study population were determined according to categories of apnea–hypopnea index (AHI). The following commonly used cut-offs for AHI were used to define 3 categories of disease severity: AHI < 15 (no OSA or mild OSA), 15 ≤ AHI < 30 (moderate OSA), and AHI ≥ 30 (severe OSA). Patients with no OSA (AHI < 5) and patients with mild OSA (5 ≤ AHI < 15) were pooled in the same group. Indeed, recent epidemiological data demonstrated that although mild OSA (5 ≤ AHI < 15) is highly prevalent, most patients are asymptomatic and the association between mild OSA and adverse clinical outcomes remains unproven [49]. For other physiologic indices of OSA severity, the study sample was grouped into quartiles of the variable. Normality of distribution was assessed using the Kolmogorov–Smirnov test. Continuous variables with normal distribution were described as mean (SD) or mean (95% confidence interval [CI]). Univariate analysis was first used to determine which primary and secondary outcomes variables could be significantly associated with OSA severity. For continuous variables, p values across AHI categories were calculated by analysis of variance. The Chi square test and the Fischer exact test as appropriate were used for categorical variables. Multivariate regression analyses were then used to characterize the independent associations between OSA severity and outcome variables. The correlation between continuous variables was assessed by Pearson’s correlation coefficient. All reported p-values were two-sided. A p value ≤0.05 was considered to indicate statistical significance. All analyses were performed using STATA^®^ version 13.1 (STATA Corp., College Station, TX, USA).

## Results

**Fig. 1:**
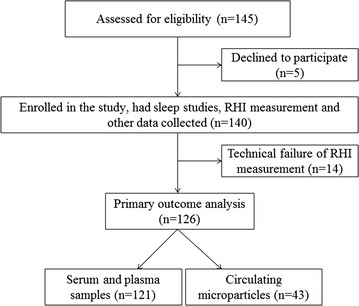
Flow diagram of subjects during the study. *RHI* reactive hyperhemia index

A flow diagram is presented in Fig. 1. Between October 12, 2011 and October 27 2015, 145 patients with T2D on stable medications and no overt CV disease were assessed for eligibility, 140 of whom consented and were enrolled in the study. Technical failure of RHI measurement occurred in 14 patients. Table 1 presents the characteristics of 126 study participants included in the primary outcome analysis according to OSA severity. Among 126 patients with T2D, 27 (21.4%) had AHI between 15 and <30 indicating moderate OSA and 60 (47.6%) had AHI ≥ 30 indicating severe OSA. Significant inter-group differences were observed only for injectable GLP-1 analog and insulin use.

**Table 1:** Characteristics of study participants according to obstructive sleep apnea severity

	All	AHI < 15	15 ≤ AHI < 30	AHI > 30	p value
n	126	39	27	60	–
Age, years	59.3 (9.5)	59.6 (10.6)	61.4 (10.4)	58.1 (8.3)	0.33
Women, %	40.3	51.3	36.0	35.0	0.24
BMI, kg/m^2^	33.2 (6.3)	32.0 (6.4)	32.5 (6.2)	34.4 (6.1)	0.14
Waist circumference, cm	113.0 (17.0)	108.0 (18.9)	115.2 (16.6)	115.3 (15.9)	0.22
Waist-to-hip ratio	1.2 (0.4)	1.1 (0.3)	1.3 (0.4)	1.3 (0.5)	0.19
Current smokers, %	13.5	18.8	14.3	7.1	0.42
Hypertension, %	59.0	46.2	66.7	64.4	0.14
Duration of diabetes, years	9.0 (7.7)	10.1 (7.4)	10.4 (8.7)	7.7 (7.3)	0.20
Metformin, %	90.3	87.1	94.7	90.7	0.83
Sulfonylurea, %	26.7	24.0	27.8	27.9	0.93
DPP-4 inhibitors, %	19.8	25.0	16.7	17.5	0.70
Injectable GLP-1 analog, %	9.6	3.7	0.0	18.0	0.01
Insulin, %	13.1	10.3	29.2	8.5	0.03
Antihypertensive medications					
ACE inhibitors, %	32.9	28.6	25.0	39.0	0.50
CCBs, %	17.1	7.7	6.3	27.5	0.05
β-blockers, %	23.2	32.1	16.7	20.0	0.38
Cholesterol-lowering drugs, %	61.4	56.7	58.8	65.9	0.71
Antiaggregants, %	14.5	18.5	11.8	12.8	0.76
Sleep-disordered breathing indices					
AHI, n	33.7 (27.5)	7.1 (4.3)	21.9 (4.0)	56.2 (23.3)	<0.0001
ODI, n	34.2 (45.7)	10.7 (17.9)	21.7 (16.6)	54.8 (56.3)	<0.0001
T90, %	54.0 (75.8)	30.8 (58.1)	35.2 (32.0)	77.9 (92.2)	<0.0001
TDS, min.	55.2 (17.6)	52.0 (16.5)	53.4 (10.2)	58.1 (20.4)	0.22

Data are expressed as mean (standard deviation) or percentages

*BMI* body mass index, *DPP-4* dipeptidyl peptidase-4, *ACE* angiotensin converting enzyme, *CCBs* calcium channel blockers, *AHI* apnea–hypopnea index, *ODI* 3% oxygen desaturation index, *T90* % sleep time spent with oxygen saturation <90%, *TDS* time desaturation summation index [(100% − mean oxygen saturation during sleep) × total sleep time] [50]

### Peripheral endothelial function and clinic blood pressure

As shown in Table 2, increasing OSA severity in patients with T2D was associated neither with a decrease in RHI value nor with an increased risk of micro-vascular endothelial dysfunction as defined by a RHI value <1.67. In a further analysis, patients were grouped into quartiles of 3% oxygen desaturation index (ODI), % sleep time spent with oxygen saturation (SaO_2_) <90% (T90), and time desaturation summation index (TDS) [(100%-mean oxygen saturation during sleep) × total sleep time] [50] in order to estimate the impact of increasing nocturnal hypoxemia on RHI in T2D patients. No significant association was observed between RHI values and increasing nocturnal hypoxemia as assessed by quartiles of ODI (p = 0.32), T90 (p = 0.33) and TDS (p = 0.85). Univariate analyses showed no significant association between RHI and additional factors including type of oral antidiabetic drug, antihypertensive medications, cholesterol-lowing medications, and antiaggregants (data no shown). No difference was observed across AHI categories regarding diastolic BP. In contrast, increasing OSA severity was associated with significantly higher systolic BP after adjustment for age, gender, body mass index (BMI), insulin and injectable GLP-1 analog use (p = 0.03).

**Table 2:** Endothelial function and clinic blood pressure (BP) according to obstructive sleep apnea severity

	AHI < 15	15 ≤ AHI < 30	AHI > 30	p value	p value*
RHI	1.84 (0.53)	1.81 (0.55)	1.84 (0.42)	0.97	–
RHI < 1.67, %	47.1	44.4	39.2	0.76	–
Systolic BP, mmHg	129.2 (12.5)	140.2 (17.9)	136.6 (18.0)	0.04	0.03
Diastolic BP, mmHg	76.6 (8.9)	78.4 (9.6)	80.9 (12.1)	0.15	–

Data are expressed as mean (standard deviation) or percentages

*RHI* reactive hyperemia index

* p values were adjusted for age, gender, body mass index, insulin and injectable GLP-1 analog use

### Biomarkers of cardiovascular risk

As shown in Table 3, increasing OSA severity was significantly associated with lower circulating levels of adiponectin (p = 0.0009) and higher levels of sP-selectin (p = 0.03) after adjusting for age, gender, BMI, insulin and injectable GLP-1 analog use. There was also a trend for a decrease in 8-isoprostane levels with increasing OSA severity but the difference across AHI categories did not reach statistical significance after adjustment for confounders. No significant differences were observed across AHI categories for other biomarkers including circulating MPs excepted for a trend toward an increase in CD62P + MPs in moderate to severe OSA (p = 0.08). A significant negative correlation was found between the HOMA-IR and the RHI (r = −0.22; p = 0.03). No correlation was observed between the RHI and other biomarkers.

**Table 3:** Biochemical variables according to obstructive sleep apnea severity

	AHI < 15	15 ≤ AHI < 30	AHI > 30	p value	p value*
HbA_1C_, %	8.0 (1.7)	7.3 (1.4)	7.4 (1.2)	0.05	–
Glucose, mmol/l	8.4 (2.2)	7.8 (2.3)	7.9 (1.9)	0.34	–
Insulin, mU/l	15.3 (11.2)	22.2 (25.0)	15.0 (10.4)	0.11	–
HOMA-IR	5.6 (4.9)	8.1 (9.5)	5.3 (3.9)	0.13	–
TC, mmol/l	4.6 (1.0)	4.6 (1.3)	4.6 (0.8)	0.99	–
HDLc, mmol/l	1.1 (0.2)	1.0 (0.2)	1.0 (0.2)	0.18	–
LDLc, mmol/l	2.7 (1.0)	2.9 (1.3)	2.7 (0.5)	0.88	–
TG, mmol/l	1.7 (0.9)	1.6 (0.6)	2.3 (1.3)	0.18	–
Adiponectin, µg/ml	14.7 (7.1)	9.2 (6.3)	9.3 (6.4)	0.0004	0.0009
Leptin, ng/ml	30.9 (20.5)	38.8 (34.2)	33.5 (25.9)	0.54	–
Leptin/adiponectin	3.5 (3.8)	5.4 (5.1)	5.5 (6.5)	0.19	–
8-isoprostane, pg/ml	401.7 (142.8)	235.7 (157.4)	304.1 (195.1)	0.01	0.09
hs-CRP, µg/ml	45.1 (60.7)	30.7 (30.7)	40.3 (35.8)	0.45	–
sP-sel, ng/ml	38.4 (17.9)	54.9 (36.9)	50.8 (25.6)	0.03	0.03
Circulating MPs (n = 43)					
CD62E^+^ MPs/ml	70.4 (45.4)	113.2 (116.5)	76.0 (66.6)	0.3882	–
CD62P^+^ MPs/250 × 10^3^platelets/ml	37.3 (22.7)	54.8 (41.2)	64.1 (37.4)	0.0838	–

Data are expressed as mean (standard deviation)

*HbA*_*1C*_ glycated hemoglobin, *HOMA-IR* homeostasis model assessment of insulin resistance, *TC* total cholesterol, *HDLc* high-density lipoprotein cholesterol, *LDLc* low-density lipoprotein cholesterol, *TG* triglycerides, *hs-CRP* high-sensitivity C-reactive protein, *sP-sel* soluble P-selectin, *MPs* microparticles

* p values were adjusted for age, gender, body mass index, insulin and injectable GLP-1 analog use

### Daytime sleepiness and quality of life questionnaires

As shown in Table 4, increasing OSA severity was associated with higher ESS (p = 0.01) although the association was attenuated after adjustment for confounders (p = 0.08). After adjustment for age, gender, BMI, insulin and injectable GLP-1 analog use, increasing OSA severity was associated lower scores in 3 domains of HRQL including energy/vitality (p = 0.02), role functioning (p = 0.01), and social functioning (p = 0.04). There was no significant association between RHI and ESS score.

**Table 4:** Daytime sleepiness and quality of life questionnaires according to obstructive sleep apnea severity

	AHI < 15	15 ≤ AHI < 30	AHI > 30	p value	p value*
Daytime sleepiness					
ESS	7.5 (4.3)	8.5 (5.3)	10.6 (5.3)	0.01	0.08
ESS ≥ 11, %	22.8	37.5	46.0	0.06	–
Quality of life					
SF36 PCS	50.4 (2.3)	50.6 (2.5)	51.0 (2.2)	0.51	–
SF36 MCS	48.1 (5.9)	47.0 (6.5)	45.4 (6.4)	0.12	–
SF36 bodily pain	60.4 (24.7)	60.9 (29.2)	54.1 (26.0)	0.41	–
SF36 energy/vitality	57.1 (20.8)	52.1 (25.9)	43.0 (23.8)	0.02	0.02
SF36 general health	65.6 (21.8)	62.3 (18.2)	56.2 (22.0)	0.11	–
SF36 mental health	67.6 (20.3)	63.5 (23.1)	57.8 (22.0)	0.09	–
SF36 physical functioning	72.3 (25.8)	65.4 (27.3)	62.5 (28.3)	0.24	
SF36 role emotional	82.1 (34.1)	76.0 (35.4)	65.1 (42.0)	0.10	–
SF36 role physical	83.3 (30.5)	66.0 (40.1)	58.0 (40.7)	0.007	0.01
SF36 social functioning	75.5 (28.7)	76.6 (25.7)	61.6 (31.1)	0.03	0.04

Data are expressed as mean (standard deviation) or percentages

*ESS* Epworth sleepiness scale, *PSQI* Pittsburgh sleep quality index, *PCS* physical component score, *MCS* mental component score

* p values were adjusted for age, gender, body mass index, insulin and injectable GLP-1 analog use

## Discussion

The main finding of the present study is the lack of additive effect of OSA severity on endothelial dysfunction in patients with T2D. Increasing OSA severity and nocturnal hypoxemia were not associated with a significant decrease in RHI. In contrast, increasing OSA severity was associated with higher systolic BP, lower circulating levels of adiponectin and higher levels of sP-selectin.

The present study confirms the very high prevalence of OSA in patients with T2D. Moderate to severe OSA prevalence in our sample of subjects with T2D (69.0%) was even higher than in previous studies [38, 39]. Among 305 obese patients with T2D, Foster et al. [39] found 53.1% of patients with moderate to severe OSA. However, the authors used the 2007 American Academy of Sleep Medicine (AASM) respiratory event criteria that were recently demonstrated to underestimate the AHI when compared to 2012 AASM criteria [51]. In a more recent study including 234 patients with T2D, 151 patients had OSA on home-based cardiorespiratory sleep study of whom only 40% had moderate to severe OSA [38]. However, a recent report from the multi-center European Sleep Apnea Cohort (ESADA) demonstrated that cardiorespiratory sleep studies interpreted using standard guidelines result in under diagnosis and misclassification of OSA [52].

Despite the high prevalence of OSA in patients with T2D, the impact of OSA severity on micro-vascular endothelial function in diabetic subjects has not been clearly elucidated. In a cross-sectional study including healthy normal-weight controls (n = 14), healthy obese controls (n = 33), subjects with T2D (n = 68), and obese subjects with OSA (n = 38), Yim-Yeh et al. [53] concluded that OSA impaired endothelial function in the brachial artery as assessed by FMD to a similar degree as T2D did. However, OSA patients compared to patients with T2D displayed a better vascular reactivity in the skin microcirculation on laser Doppler flowmetry after acetylcholine iontophoresis. This study was not designed to evaluate an additive effect of OSA severity on endothelial dysfunction. Furthermore T2D patients had no sleep study and it is likely that many of these patients had undiagnosed OSA. More recently, Tahrani et al. [38] studied micro-vascular function by laser speckle contrast imaging in patients with T2D with OSA (n = 47) and without OSA (n = 24). After adjustment for confounders, OSA was associated with impairments in basal micro-vascular flux and endothelial-independent micro-vascular reactivity (sodium nitroprusside) but not with endothelial-dependent micro-vascular reactivity (acetylcholine). We acknowledge the lack of healthy control group in our study. In a recent study including 53 healthy subjects with a mean BMI of 25 (4.3) kg/m^2^, the mean RHI value was 2.4 (0.6) [23]. In a recent randomized controlled trial including 150 patients with severe OSA (median [interquartile range] AHI, 41.0 [35.0–53.0]; mean BMI, 27.0 (3.2) kg/m^2^), with no overt CV disease and only 5% of comorbid TD2, the mean RHI value was 2.15 (0.61) [54]. The mean RHI value in our population of diabetic subjects was markedly lower [≈1.8 (0.5)] with no difference across AHI categories. Altogether, our findings and those of previous studies suggest that TD2 has major adverse effects on endothelial function and that endothelial dysfunction could not be made any worse by OSA. The lack of difference in MPs from activated endothelial cells (CD62E + MPs) across AHI categories also reinforced the notion that OSA does not aggravate endothelial dysfunction in T2D patients. This result is consistent with findings of a recent study demonstrating the lack of association between OSA and endothelial function assessed by RHI in obese subjects [55]. Endothelial dysfunction is linearly associated with dysglycemia even in the absence of overt diabetes [56, 57]. Our results are also partially consistent with findings of recent randomized trials showing that CPAP therapy of OSA has no effect on glycemic control in patients with TD2 [58], and does not prevent CV events in patients with established CV disease [59].

Oxidative stress and concomitant systemic inflammation are two of the prominent underlying mechanisms suggested to contribute to the increased risk for CV disease both in OSA [60] and T2D [61]. In the present study, there was a trend for a decrease in 8-isoprostane levels with increasing OSA severity although the inter-group difference did not reach statistical significance after adjustment for confounders. Despite the general belief that OSA increases oxidative stress, a recent randomized controlled study has demonstrated a reduction in urinary F2-isoprostane after 2 weeks of intermittent hypoxia following continuous positive airway pressure (CPAP) withdrawal [62]. CPAP withdrawal was also associated with a rise in plasma superoxide dismutase, an antioxidant known to be increased by hypoxic preconditioning. Paradoxical data from epidemiological mortality endpoint studies also supported the existence of protective preconditioning effects in OSA [60]. Further studies are required to investigate potential protective effects of OSA against oxidative stress in T2D. Among various adipokines, adiponectin has attracted considerable attention due to its role in CV disorders. It has been suggested that reduced serum adiponectin levels could partly explain increased CV disease in OSA patients [63]. Interestingly, increasing OSA severity in our sample of patients with T2D was associated with a significant reduction in adiponectin levels after adjustment for confounding factors including BMI. P-selectin is an adhesion molecule that is expressed on activated platelets and endothelium and is shed into plasma in a soluble form, sP-selectin [64]. P-selectin is involved in leukocyte rolling and attachment, and thus can play an important role in the initiation of atherosclerosis [65]. Serum levels of sP-selectin were found to be increased in moderate to severe OSA patients and were associated with a higher prevalence of silent brain infarction on brain magnetic resonance images [66]. In the present study, moderate to severe OSA was associated with elevated levels of sP-selectin in patients with T2D. There was also a trend toward an increase in MPs derived from activated platelets (CD62P^+^) in moderate to severe OSA. Although we failed to demonstrate a significant correlation between RHI, adiponectin and sP-selectin, both might contribute to increase the risk of CV events in T2D patients with moderate to severe OSA.

Our study has some strengths and limitations. The major strengths include the availability of PSG data, which is the gold standard for OSA diagnosis, and endothelial function assessment in a relatively large sample of patients. The major limitation of our study is its cross-sectional design and the lack of an interventional arm. Further studies are required to determine whether low RHI values predict poorer CV outcomes in patients with OSA and T2D. In the present study we investigated peripheral endothelial function at the level of the arterioles, which are most importantly related to peripheral vascular resistance [67, 68]. Although changes in pulse wave amplitude during reactive hyperemia were found to be correlated with brachial artery FMD [11, 12], the NO-mediated component totals ≈60% of digital artery dilation, whereas the remainder represents other vasodilator components [69]. It cannot be excluded that the findings would have been different if we used a different method for vascular function assessment. For example nocturnal non-dipping, a typical BP pattern in OSA [70], has been found to be associated with nitroglycerin-mediated vasodilation but not with FMD [71]. However, there is strong evidence that PAT is a reliable and reproducible technique to accurately characterize endothelial function in multicenter studies [3–7, 72]. A recent review [3] suggested that micro-vascular function might be more relevant in subjects without overt CV disease and may be an earlier indicator of CV risk than macrovascular function assessed by FMD. From a technical viewpoint, PAT offers the advantages of being less prone to operator error than FMD [69]. We acknowledge that we did not evaluate fluctuations in glucose levels by continuous glucose monitoring in the present study. Indeed, fluctuations in blood glucose levels were found to play a significant role in vascular endothelial dysfunction assessed by RHI in TD2 [73]. Nocturnal hyperglycemia has been reported in patients with TD2 and OSA particulary during REM sleep apneas with marked oxygen desaturations [74]. However, no significant association was observed between RHI values and increasing nocturnal hypoxemia in the present study.

## Conclusion

Moderate to severe OSA is very common but has no impact on digital micro-vascular endothelial function in patients with T2D.

## Abbreviations

AHI: apnea–hypopnea index
BMI: body mass index
BP: blood pressure
CV: cardiovascular
ESS: Epworth sleepiness scale
FMD: flow-mediated dilatation
HbA1c: glycated hemoglobin
HDLc: high-density lipoprotein serum cholesterol
HOMA-IR: homeostasis model assessment resistance index
HRQL: health related quality of life
LDLc: low-density lipoprotein serum cholesterol
NO: nitric oxyde
ODI: 3% oxygen desaturation index
OSA: obstructive sleep apnea
PAT: peripheral arterial tonometry
PSQI: Pittsburgh sleep quality index
PSG: polysomnography
T2D: type 2 diabetes
T90: % sleep time spent with oxygen saturation (SaO_2_) <90%
RHI: reactive hyperemia index
SF36: medical outcomes study 36-item short-form
